# Supplementary material for: Sicilian Rivet Wheat Landraces: Grain Characteristics and Technological Quality of Flour and Bread
Source: Plants (Basel). 2023 Jul 14;12(14):2641. doi: 10.3390/plants12142641 (PMC10385672; doi:10.3390/plants12142641)
Supplement: Supplementary file 1 [file plants-12-02641-s001.zip › plants-2456760-supplementary.pdf]

Table S1 - K-means Cluster Analysis. Cluster membership.

Cluster Membership

Case Number	SAMPLE	Cluster	Distance
1	Bidì 03	1	3.518
12	Bufala Rossa Corta b01	1	3.518
2	Simeto	2	0.000
7	Bufala Nera Corta 01	3	4.463
13	Bufala Rossa Lunga 01	3	3.871
18	Ciciredda 02	3	3.203
9	Bufala Nera Lunga 01	4	3.171
10	Bufala Nera Lunga 02	4	2.792
14	Bufala Rossa Lunga 03	4	4.129
3	Bivona 03	5	4.212
4	Bivona 04	5	4.399
5	Bufala Bianca 02	5	4.098
6	Bufala Bianca 03	5	5.475
8	Bufala Nera Corta 02	5	4.973
11	Bufala Nera Lunga 04	5	5.013
15	Bufale Cerami 01	5	4.488
16	Bufale Salice 01	5	5.029
17	Bufale Troina 01	5	4.081
19	Ciciredda 03	5	3.616
20	Paola 02	5	3.342

Table S2 - K-means Cluster Analysis. ANOVA results.

ANOVA

	Cluster		Error			
	Mean Square	df	Mean Square	df	F	Sig.
Zscore: P/L	4.351	4	.106	15	40.854	.000
Zscore: W (10^{-4} x J)	4.313	4	.117	15	36.985	.000
Zscore: Semolina_b*	4.275	4	.127	15	33.748	.000
Zscore: H_2O absorpion in 500 B.U.) (%)	4.086	4	.177	15	23.061	.000
Zscore: Dough stability (min)	3.917	4	.222	15	17.644	.000
Zscore: Dry gluten	3.762	4	.264	15	14.276	.000
Zscore: Development time (min)	3.710	4	.277	15	13.385	.000
Zscore: Bread_weight (g)	3.457	4	.345	15	10.028	.000
Zscore: Starchy kernels (%)	3.442	4	.349	15	9.870	.000
Zscore: Bread_Volume (cm^3)	3.385	4	.364	15	9.297	.001
Zscore: Peak Dough Height (M.U.)	3.183	4	.418	15	7.621	.001
Zscore: Degree of softening	3.178	4	.419	15	7.580	.002
Zscore: Semolina_L*	3.067	4	.449	15	6.832	.002
Zscore: SDS sedimentation height (mm)	3.001	4	.466	15	6.436	.003
Zscore: Wet gluten	2.982	4	.471	15	6.326	.003
Zscore: Ash (g/100g d.m.)	2.707	4	.545	15	4.968	.009
Zscore: Protein (g/100g d.m.)	2.706	4	.545	15	4.965	.009
Zscore: Crumb_yellow index (b*)	2.607	4	.572	15	4.560	.013
Zscore: Hectoliter weight (kg/hL)	2.535	4	.591	15	4.291	.016
Zscore: Bread_height (mm)	2.504	4	.599	15	4.180	.018
Zscore: Water Binding in wet Gluten (%)	2.401	4	.626	15	3.833	.024
Zscore: Semolina_a*	2.360	4	.637	15	3.704	.027
Zscore: Thousand kernel weight (g)	2.223	4	.674	15	3.300	.040
Zscore: Gluten Index	2.069	4	.715	15	2.895	.058
Zscore: Shrunken kernels (%)	2.024	4	.727	15	2.784	.065

Zscore: Mixing Time (s)	1.890	4	.763	15	2.477	.089
Zscore: Crust_red index (a*)	1.679	4	.819	15	2.051	.139
Zscore: Bread_moisture (%)	1.617	4	.836	15	1.935	.157
Zscore: Crust_yellow index (b*)	1.570	4	.848	15	1.851	.172
Zscore: Moisture (g/100 g)	1.270	4	.928	15	1.368	.292
Zscore: Crust_L*	1.064	4	.983	15	1.082	.400
Zscore: Bread_Porosity (1-8)	1.006	4	.999	15	1.007	.435
Zscore: Falling number (s)	.875	4	1.033	15	.846	.517
Zscore: Crumb_red index (a*)	.284	4	1.191	15	.238	.912
Zscore: Black point (%)	.184	4	1.218	15	.151	.960
Zscore: Crumb_L*	.107	4	1.238	15	.086	.985

The F tests should be used only for descriptive purposes because the clusters have been chosen to maximize the differences among cases in different clusters. The observed significance levels are not corrected for this and thus cannot be interpreted as tests of the hypothesis that the cluster means are equal.
